# Ultrasound-guided erector spinae plane block for perioperative analgesia in patients undergoing laparoscopic nephrectomies surgery: a randomized controlled trial

**DOI:** 10.1186/s13063-023-07866-0

**Published:** 2024-01-02

**Authors:** Ming Yang, Lei Cao, Tong Lu, Cheng Xiao, Zhuoxi Wu, Xuetao Jiang, Wei Wang, Hong Li

**Affiliations:** Department of Anesthesiology, Xinqiao Hospital of Chongqing, Second Affiliated Hospital of Army Medical University, PLA, Chongqing, 400037 China

**Keywords:** Erector spinae plane block, Dexmedetomidine, Dexamethasone, Nephrectomy

## Abstract

**Background:**

Laparoscopic nephrectomy is a commonly utilized surgical approach for the management of renal cancer. Despite its widespread acceptance, postoperative pain management remains a significant challenge for many patients undergoing this procedure. Traditional pain management techniques, including opioid and nonsteroidal anti-inflammatory drug administration, may not provide adequate pain relief and may result in adverse effects. In recent years, erector spinae plane block (ESPB) has emerged as a promising regional anesthesia technique due to its simplicity, safety, and potential efficacy in reducing postoperative pain. ESPB has demonstrated effectiveness in reducing postoperative pain in various surgical procedures. However, the efficacy of ESPB in laparoscopic nephrectomy for renal cancer has not been extensively studied. As such, further investigation is necessary to determine the potential benefits of ESPB in this context. The addition of adjuvants such as dexmedetomidine and dexamethasone to nerve blocks has been shown to improve both the duration and quality of the block. Multiple studies have demonstrated the safety and efficacy of these adjuvants in reducing postoperative pain and opioid consumption and improving patient satisfaction. The use of dexmedetomidine and dexamethasone as adjuvants for nerve blocks represents a promising approach for enhancing regional anesthesia and analgesia. In light of these findings, we have incorporated dexmedetomidine and dexamethasone into our nerve block protocol.

**Methods:**

This study is a randomized controlled trial conducted at a single center, with 50 participants being randomized in a 1:1 ratio to either the ESPB group or the control group. The trial aims to investigate the efficacy of ESPB in patients diagnosed with kidney cancer who are scheduled for laparoscopic nephrectomy. The primary outcome measure is the total consumption of intraoperative sufentanil. Secondary outcomes include the VAS score at rest and during coughing at 1 h, 6 h, 12 h, 24 h, and 48 h after surgery; total intraoperative remifentanil consumption; the number of times rescue analgesia is required; and the incidence of nausea and vomiting in the first 24 h after surgery. This study is registered for a duration of 1 year and is being conducted in China.

**Discussion:**

The objective of our study is to evaluate the potential benefits of erector spinae plane block (ESPB) in patients undergoing laparoscopic nephrectomy, with a focus on the impact of dexmedetomidine and dexamethasone as adjuvants on the quality and duration of the block, as well as postoperative pain and opioid consumption. By investigating the effects of these adjuvants in the context of ESPB, we hope to contribute to the growing body of literature on the use of adjuvants in nerve blocks and provide further insight into the potential benefits of this approach for improving patient outcomes following laparoscopic nephrectomy. This trial was approved by the Ethics Committee of the Second Affiliated Hospital of Army Medical University.

**Trial registration:**

China Clinical Trial Register ChiCTR2300068578. Registered on 20 February 2023.

## Administrative information

**Table:** 

Title {1}	Ultrasound-guided erector spinae plane block for perioperative analgesia in patients undergoing laparoscopic nephrectomies surgery: a randomized controlled trial
Trial registration {2a and 2b}	Clinical Trial Register ChiCTR2300068578. Registered on 20 February 2023
Protocol version {3}	Version 1.0 of 20 February 2023
Funding {4}	The study was funded by the National Natural Science Foundation of China (Project No.82171265), the National Key Research and Development Program of China (Project No. 2018YFC0117200) and others.
Author details {5a}	Department of Anesthesiology, Xinqiao Hospital of Chongqing, Second Affiliated Hospital of Army Medical University, PLA, Chongqing 400037, China
Name and contact information for the trial sponsor {5b}	Name: Ke Wang Address: Department of Anesthesiology, Xinqiao Hospital of Chongqing, Second Affiliated Hospital of Army Medical University, PLA, Chongqing 400037, China. Email: ke.wang@oist.jp
Role of sponsor {5c}	The sponsor will take part in neither the process of the trial nor the decision to submit the results.

## Introduction

### Background and rationale {6a}

Kidney cancer has become increasingly prevalent and is now ranked among the top ten most common cancers [1]. The most commonly used treatment method for kidney cancer is radical or partial nephrectomy [2]. However, with the rise of minimally invasive surgical techniques, such as laparoscopic and robot-assisted nephrectomy, these methods have become more widely used in clinical practice. Although they bring less trauma to patients, moderate to severe pain remains a common issue in laparoscopic nephrectomy [3]. Concurrently, the concept of enhanced recovery after surgery (ERAS) has gained popularity due to its evidence-based practices that aim to reduce hospitalization lengths and improve the quality of patient recovery [4]. Adequate pain control plays a critical role in ERAS, and multimodal analgesia, including the regional nerve block approach, has been proposed as an analgesic option for patients undergoing laparoscopic nephrectomy.

Erector spinae plane block (ESPB) is a relatively new block technique that was developed by Forero et al. and has been proposed as an alternative analgesic approach in various surgeries, including spinal, gallbladder, liver, and cardiac surgery [5–8]. ESPB has gained popularity due to its relative simplicity and safety when compared to paravertebral block. In laparoscopic nephrectomy, dissection of the retroperitoneal space can lead to visceral and somatic pain caused by the T6–T12 somatic nerves [9]. A recent case report demonstrated that ESPB could provide postoperative analgesia in laparoscopic nephrectomy [10]. However, randomized controlled clinical trials are limited in this area. Therefore, this study will aim to assess the effect of ESPB combined general anesthesia vs general anesthesia alone on pain control in patients undergoing laparoscopic nephrectomy.

### Objectives {7}

We have designed a prospective, randomized, double-blinded experiment to investigate the hypothesis that ESPB may provide superior perioperative analgesia in patients undergoing scheduled laparoscopic nephrectomy.

### Trial design {8}

**Fig. 1:**
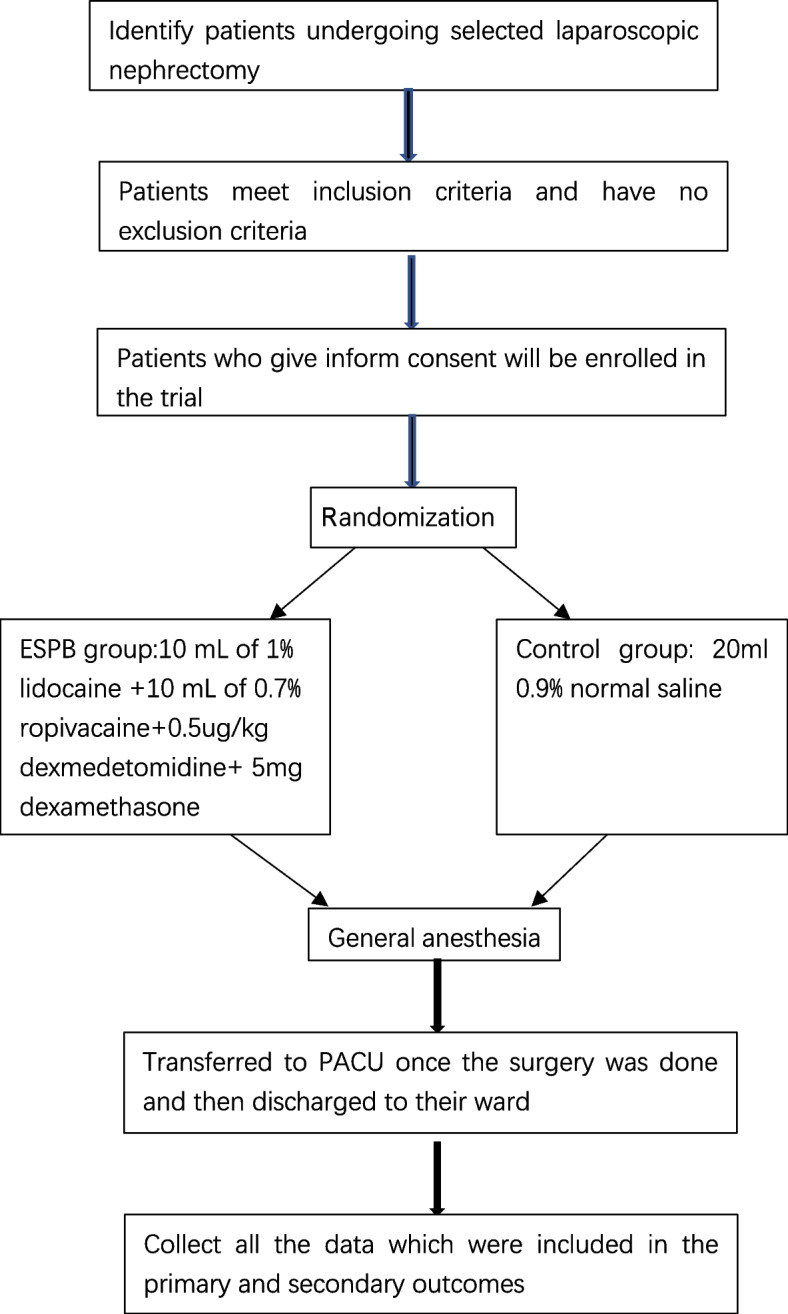
Study flow chart

This is a randomized, double-blind, single-center controlled trial conducted at the Second Affiliated Hospital of Army Medical University, PLA. The study flow chart is presented in Fig. 1. Patients undergoing scheduled laparoscopic nephrectomy will be recruited and randomized in a 1:1 ratio to receive either ESPB or standard pain management. Both groups will receive a standardized pain relief protocol during and after surgery. Participant enrollment began in February 2023 and will continue until February 2024. Our research was supported by grants from the National Natural Science Foundation of China (Project No.82171265) and the National Key Research and Development Program of China (Project No.2018YFC0117200).

## Methods: participants, interventions, and outcomes

### Study setting {9}

The study was approved by the Ethics Committee of the Second Affiliated Hospital of Army Medical University and registered on the Chinese Clinical Trial Registry (number: ChiCTR2300068578). Written informed consent will be obtained from each individual prior to enrollment.

### Eligibility criteria {10}

Individuals meeting all of the following criteria will be included:

1. Patients aged 18–65 years scheduled for elective laparoscopic nephrectomy
2. ASA I–III
3. Willing to participate in this study and signed informed consent

Patients who meet one of the following criteria will be excluded:

1. Allergy to local anesthetics.
2. BMI > 35 kg/m^2^.
3. History of mental illness or taking psychotropic drugs.
4. Combined with spinal deformity.
5. Long-term use of analgesics or sedatives.
6. History of drug abuse, alcohol, or opioid abuse.
7. Chronic pain or recent acute pain.
8. Severe liver and kidney dysfunction.
9. Participants have the right to withdraw from the study at their discretion for any reason or if the researchers determine that their inclusion in the trial is not appropriate.

### Who will take informed consent? {26a}

Enrollment of eligible participants into the study will be overseen by a designated member of the research team. Informed consent, as approved by the Institutional Review Board, will be obtained from eligible patients or their legally authorized representative, after a thorough verbal explanation of the study and its associated benefits and risks. Participants will be informed that their participation is entirely voluntary and that they may withdraw from the trial at any time.

### Additional consent provisions for collection and use of participant data and biological specimens {26b}

Not applicable. We will not use the data and biological specimens of participants in ancillary studies.

### Interventions

#### Explanation for the choice of comparators {6b}

To the best of our knowledge, there is a dearth of prospective clinical studies investigating the efficacy of ESPB in patients undergoing minimally invasive renal surgery. As such, there is a need for further research to elucidate the impact of ESPB on postoperative pain management and to establish optimal regional analgesic techniques for this specific type of surgery. Given the potential benefits of ESPB in reducing opioid consumption and minimizing adverse effects associated with systemic analgesics, such research is of paramount importance in advancing the field of perioperative pain management.

#### Intervention description {11a}

The ESPB procedure was performed before the induction of general anesthesia in the operating room. After giving 0.2 mg/kg midazolam and 0.1 μg/kg sufentanil, subjects were placed in a lateral position under sterile conditions. High-frequency linear array probe and a 21-gauge nerve block needle were used.

Upon positioning patients in a lateral position, the T8 spinous process was identified on the midline of the spine using a linear probe. Subsequently, the ultrasound probe was moved 2–3 cm laterally to locate the hyperechogenicity of the T8 transverse process and identify the erector spinae muscle. Using an in-plane technique, a 21-G needle was advanced in a cranio-caudal direction until the tip of the needle reached the tip of the T8 transverse process. The correct position of the needle tip was confirmed by hydrodissection of the interfascial plane using 2–3 ml of 0.9% normal saline. Under ultrasound guidance, a solution comprising 10 ml of 1% lidocaine, 10 ml of 0.7% ropivacaine, 0.5 μg/kg dexmedetomidine, and 5 mg dexamethasone was injected, and the spread of local anesthetics between the transverse processes and the erector spinae muscle was observed in real time. For the control group, the same procedure was performed, and patients were injected with 20 ml of 0.9% normal saline.

#### Criteria for discontinuing or modifying allocated interventions {11b}

The withdrawal criteria are as follows:

1. Patient request to withdraw from the study.
2. A change in the surgical procedure may occur during surgery.
3. The investigator may decide to end the trial due to other unforeseen reasons.

#### Strategies to improve adherence to interventions {11c}

Our trial will be closely monitored by an independent investigator to ensure that all procedures are consistent with the approved protocol. Additionally, the randomization process will be performed by another investigator to ensure impartiality and minimize the potential for bias. All experiments will be conducted strictly in accordance with the study protocol to maintain consistency and ensure the reliability and validity of our findings.

#### Relevant concomitant care permitted or prohibited during the trial {11d}

Our study will involve comprehensive monitoring of participants, including electrocardiographs, noninvasive blood pressure, and pulse oxygen saturation. In addition, depth of anesthesia monitoring will be routinely utilized in all subjects to ensure optimal anesthetic management. Participants will be induced with propofol, sufentanil, and rocuronium, followed by tracheal intubation. General anesthesia will be maintained using propofol (2–4 mg/kg), remifentanil (0.1–0.2 μg/kg/min), and sevoflurane (1–2%) to achieve a target BIS score between 40 and 60. All participants will receive a fluid infusion of 5–7 ml/kg (ringer acetate or compound electrolytes and solution). Remifentanil infusion rate will be adjusted to maintain patients’ arterial blood pressure and heart rate changes no more than 20% of baseline values. Volume control ventilation will be selected for intraoperative ventilation management, with ventilation parameters set as follows: tidal volume (6–8 ml/kg, ideal body weight), I:E ratio (1:2), respiratory rate (12–14 bpm), positive end-expiratory pressure (PEEP 5–8 cmH_2_O), and fraction of inspired oxygen (0.40). Tropisetron (5 mg, i.v.) will be administered half an hour before the end of surgery to prevent nausea and vomiting. Following surgery, all patients will be transferred to the post-anesthesia care unit (PACU) and extubated when they meet the criteria for extubation.

Once the patient is transferred to PACU, a transvenous patient-controlled analgesia (PCA) pump will be connected. Patients will be assessed for pain intensity by an anesthesia nurse with a visual analog scale (VAS) 10 min after extubation. Dezocine (5 mg i.v.) will be used as a rescue analgesia for pain score > 3/10 at rest or with a cough. The patient will be reassessed for pain 10 min after dosing. Another dose of dezocine (5 mg i.v.) will be given if the score is still more than 3/10. The PCA pump is formulated as 3 μg/kg sufentanil and 5 mg tropisetron mesylate diluted to 180 ml with 0.9% saline. The parameters of analgesia pump are as follows: a loading dose of 2 ml, a background dose of 4 ml/h, a bolus controlled of, and a lock time of 15 min. All patients will be taught how to use a PCA pump.

#### Provisions for post-trial care {30}

In the event that trial participants experience any complications resulting from the interventions, they will receive standard post-operative management from a multidisciplinary team consisting of the surgical team, anesthesiologist team, and even care team. Our team is dedicated to providing fine-tuned management for all participants to ensure that any adverse events are promptly identified and appropriately managed. Additionally, we will closely monitor participants throughout the post-operative period to identify any potential complications and provide timely interventions as needed. Our goal is to ensure that all participants receive the highest level of care and support throughout the trial.

### Outcomes {12}

#### Primary outcome

The primary outcome is the total consumption of intraoperative sufentanil.

#### Secondary outcomes

The following are the secondary outcomes:

1. VAS pain scores at rest at 1 h, 6 h, 12 h, 24 h, and 48 h after surgery
2. VAS pain scores at coughing at 1 h, 6 h, 12 h, 24 h, and 48 h after surgery
3. Total intraoperative remifentanil consumption
4. Times of rescue analgesia
5. The incidence of nausea and vomiting in the first 24 h after surgery

### Participant timeline {13}

The schedule of enrollment, interventions, and assessments is shown in Fig. 1.

### Sample size {14}

The primary outcome measure for this study is the total consumption of intraoperative sufentanil. To determine the appropriate sample size, we conducted a pre-test in which we measured the total amount of sufentanil used during the intraoperative period in two groups (ESPB group: 32.5 ± 5.2, control group: 38 ± 5.3) with 10 patients in each group. Based on these results, we calculated the required sample size assuming a type I error protection of 0.05, a type II error of 0.1, and a dropout rate of 20%. We use the medical software of medsci to calculate the sample size. Our analysis indicated that a sample size of 25 participants per group will provide 90% power to detect a statistically significant difference between the groups. By utilizing this sample size, we aim to ensure that our study is adequately powered to detect meaningful differences in the consumption of intraoperative sufentanil between the two groups

### Recruitment {15}

The principal investigator will be responsible for all aspects of the recruitment process and amendment.

### Assignment of interventions: allocation

#### Sequence generation {16a}

In this study, participants will be enrolled and randomly allocated to either the ultrasound-guided ESP block (ESPB) group or the control group using a computerized random-number generator (https://www.sealedenvelope.com) in a 1:1 ratio. The randomization process will be performed by an independent individual who is not involved in the study to ensure that allocation is unbiased and free from potential confounding factors. Randomization is a crucial component of the study design, as it helps to minimize selection bias and ensure that any observed differences between the groups are attributable to the intervention being studied. By utilizing a computerized randomization process and an independent individual to perform the allocation, we aim to ensure that our study is conducted with the highest level of scientific rigor and integrity.

#### Concealment mechanism {16b}

To ensure the integrity of the randomization process, patient codes and group allocation will be placed in closed, opaque envelopes, and the random sequence will be blinded to the researchers. This approach helps to minimize the potential for selection bias and ensures that the allocation of participants to the study groups is unbiased. During the follow-up process, random codes will be used to analyze the data, further ensuring that the researchers remain blinded to the group allocation.

#### Implementation {16c}

Prior to the induction of general anesthesia, the integrity of the randomization process will be confirmed by opening the opaque envelopes containing the group allocation for each participant. This process will be performed by the operator and will ensure that the allocation is unbiased and free from potential confounding factors. To further ensure the validity of the study, the participant’s signed informed consent will be obtained before the opening of the opaque envelopes.

### Assignment of interventions: blinding

#### Who will be blinded {17a}

Due to the nature of the trial, it will not be possible to blind the treating anesthesiologist to the group allocation. However, to minimize the potential for bias, all patients included in the study will be blinded to their group assignment. Additionally, the analyzing statisticians and postoperative follow-up staff will be blinded to group allocation to ensure that the data analysis is unbiased and free from potential sources of confounding factors.

#### Procedure for unblinding if needed {17b}

Group allocation will be revealed if the participant meets the criteria for terminating the experiment.

### Data collection and management

#### Plans for assessment and collection of outcomes {18a}

Data for this study will be collected from electronic medical records systems or case report forms (CRFs). The data collection process will be performed by investigators who are independent of the group allocation to minimize the potential for bias. Prior to data collection, all investigators will receive training from the principal investigator on how to collect, record, and store the data. To ensure the confidentiality of the data, all information will be kept strictly confidential and used solely for research purposes. After data collection, the data will be entered into Microsoft’s Excel system by the same investigator, and the principal researcher will thoroughly check for any missing critical data points or errors in the raw data sheets. The principal researcher will remain blinded to group allocation until the completion of data analysis to minimize the potential for bias and ensure the validity of the study findings. By utilizing this approach to data collection and analysis, we aim to ensure that our study is conducted with the highest level of scientific rigor and integrity.

#### Plans to promote participant retention and complete follow-up {18b}

In this clinical trial, a comprehensive postoperative follow-up period of 2 days will be implemented for all participants. Prior to obtaining informed consent, participants will receive detailed information regarding the study protocol and procedures to ensure full understanding and cooperation. Our research team is dedicated to addressing any unforeseen issues that may arise during the follow-up period, including patient discomfort or complications, in order to promote complete and accurate postoperative assessments. Additionally, we will employ proactive measures, such as regular check-ins and communication with participants, to further enhance patient compliance and minimize the risk of loss to follow-up.

#### Data management {19}

All participants’ data will be recorded in the CRFs. Then, all data will be transcribed onto Microsoft Excel by two investigators with double-checking. This electronic data will be stored on a computer with dual password protection. An authorized person will have access to the files.

#### Confidentiality {27}

The patients will remain enrolled throughout the study. All research data will be coded by an identification number and stored in a secure cabinet throughout the trial to guarantee confidentiality.

#### Plans for collection, laboratory evaluation, and storage of biological specimens for genetic or molecular analysis in this trial/future use {33}

Not applicable, no samples will be collected.

## Statistical methods

### Statistical methods for primary and secondary outcomes {20a}

All data will be performed with the SPSS software version 26.0 (IBM, New York, USA). A *P* value < 0.05 will be considered statistically significant. All normally distributed continuous variables will be expressed as mean ± standard deviation and analyzed with Student’s *t* test and a Mann-Whitney *U* test for non-normally distributed data and ANOVA for repeated measured data. Categorical variables were described with frequencies (%) and compared with the chi-square test or Fisher’s exact test.

### Interim analyses {21b}

Interim analysis is not currently planned.

### Methods for additional analyses (e.g., subgroup analyses) {20b}

Additional analyses are not planned.

### Methods in analysis to handle protocol non-adherence and any statistical methods to handle missing data {20c}

According to our study protocol design, we have implemented measures to minimize the risk of data loss. However, in the event that missing data cannot be ignored, our research team will utilize multiple imputation techniques to accurately estimate the missing values.

### Plans to give access to the full protocol, participant-level data, and statistical code {31c}

In this study, all data analysis will be conducted in a rigorous and transparent manner to ensure the accuracy and reliability of our findings. The statistical code used in our analyses will be made available to interested parties upon reasonable request from the principal investigator. This will allow for greater transparency and reproducibility of our analyses, and facilitate further research and collaboration in the field.

Furthermore, we will retain all completed data for a period of 5 years following the completion of the study. This is in accordance with standard data retention policies and will allow for future validation and verification of our findings. All data will be securely stored and maintained in compliance with relevant data protection laws and regulations.

### Oversight and monitoring

#### Composition of the coordinating center and trial steering committee {5d}

This clinical trial will be conducted as a single-center study, with all participants receiving care and treatment at a single research site. To ensure the smooth and effective implementation of the trial, a trial steering committee composed of four experienced clinical researchers will be established. The committee will be responsible for overseeing the trial progress on a monthly basis, reviewing all data, and addressing any issues or concerns that may arise throughout the trial period.

The trial steering committee will play a critical role in ensuring the integrity and quality of the study, providing guidance and support to the research team, and ensuring that all trial procedures are conducted in accordance with ethical and regulatory standards. The committee will also be responsible for monitoring participant safety and well-being and for making any necessary adjustments to the trial protocol to ensure the best possible outcomes for all participants.

#### Composition of the data monitoring committee, its role, and reporting structure {21a}

No data monitoring committee will be set up in our trial as the sample size is not too large. However, our center will conduct data audits three times a year. Data collection will be accomplished in 6 to 12 months.

#### Adverse event reporting and harms {22}

ESPB is a well-established technique and has been safely applied in clinical. There are no anticipated issues that could be harmful to participants. However, detrimental or unpredictable side effects will be recorded and reported, then the study will be terminated.

#### Frequency and plans for auditing trial conduct {23}

As our trial is not supported by a research budget, we have implemented an auditing process to ensure the accuracy and integrity of our data. The auditing process will involve a thorough review of all study data, including verification of missing data, confirmation of the accuracy and quality of original data, and consultation on the overall progress of the study. To ensure the independence and objectivity of the auditing process, an independent staff member who is not involved in the study will be responsible for conducting the audit. This staff member will have expertise in data auditing and will be trained on the specific requirements and procedures of our trial.

#### Plans for communicating important protocol amendments to relevant parties (e.g., trial participants, ethical committees) {25}

Our study protocol was approved by the Ethics Committee of the Second Affiliated Hospital of Army Medical University and the Chinese Clinical Trial Registry. Any amendment of the protocol will be agreed upon by the principal investigator, then re-checked and sought approval by both of them.

#### Dissemination plans {31a}

Upon the conclusion of the trial, the findings will be disseminated through peer-reviewed scholarly journals and presented at international academic conferences. Our study aims to enhance the current understanding of perioperative analgesia management and offer clinicians and patients a broader range of options for pain relief.

## Discussion

This prospective, randomized, single-center controlled study aims to evaluate the efficacy of erector spinae plane block in conjunction with general anesthesia versus general anesthesia alone for perioperative pain management in patients undergoing selected laparoscopic nephrectomy. Additionally, the study seeks to investigate the duration of using ropivacaine and adjuvants in ESPB.

The erector spinae plane block (ESPB) involves the injection of local anesthetics into the erector spinae muscle and the tips of the transverse process, making it an inter-fascial block [11]. Clinical studies have shown that the ESPB not only targets the dorsal and ventral branches but also sympathetic fibers, providing both visceral and somatic analgesia [12]. The primary mechanism of action for the ESPB is the physical spread of local anesthetics into the paravertebral space [5]. This occurs through the branches of the dorsal rami and accompanying blood vessels that perforate the posterior thoracolumbar fascia and intertransverse connective tissue complex [13, 14]. However, this spread is gradual rather than rapid, with only a small portion of the local anesthetics entering the paravertebral space during the first 30–60 min, while most remain in the erector spinae plane [15, 16]. Other proposed mechanisms of action for the ESPB include local anesthetic absorption through the fascia and systemic absorption, as well as immunomodulatory analgesia through the lymphatic system [11].

Dexmedetomidine is a highly selective alpha-2 receptor agonist that exhibits sedative and adjunctive analgesic effects. Recent studies have shown that dexmedetomidine can be used as an adjunct to local anesthetics to prolong the duration of peripheral nerve block [17]. Clinical trials have demonstrated that the addition of perineural dexmedetomidine (100 μg) to ropivacaine can double the duration of an ulnar nerve block compared to ropivacaine alone [18]. Another study has also shown that perineural administration of 0.5 μg/kg dexmedetomidine with ropivacaine can prolong the analgesic duration and reduce opioid consumption [19]. Furthermore, Vorobeichik et al. conducted a meta-analysis of 32 trials and found that perineural dexmedetomidine can improve the quality of regional nerve block by extending both sensory and motor block durations and improving onset times [17]. The dexmedetomidine-related adverse effects observed, including bradycardia and hypotension, were transient, reversible, and did not require any intervention. The authors suggested that a dose of 50–60 μg of dexmedetomidine could maximize sensory blockade while minimizing cardiovascular side effects.

Dexamethasone, a potent and long-lasting glucocorticoid, has been demonstrated to be an effective adjuvant to local anesthetics (LAs) in prolonging regional anesthesia. Combining dexamethasone with medium and short-acting LAs has been shown to increase the mean analgesic time by 4 h, while combining it with long-acting LAs can extend the mean analgesic time by up to 8 h [20]. Additionally, a meta-analysis has shown that the administration of perineural dexamethasone with LAs can slightly prolong the duration of analgesia [21]. In our study, we aim to simultaneously administer dexmedetomidine and dexamethasone with LAs in order to further enhance the duration of analgesia. By combining these two adjuvants, we hope to achieve a more significant and prolonged effect on regional anesthesia.

We have some limitations in our study. As the blocking will be performed in the operating room, we cannot avoid the bias from the anesthesiologist who will participate in this procedure. Moreover, the optimal dose and duration of these adjuvants for ESPB need further investigation.

## Trial status

The trial was registered on ClinicalTrials.gov Identifier: ChiCTR2300068578. The trial protocol Ver.1 was approved on 23 February 2023. The ethics approval was released on 20 February 2023. The trial recruitment was initiated on 24 February 2023. We are currently conducting this investigation and expect to complete this work by August.

## Abbreviations

ERAS: Enhanced recovery after surgery
ESPB: Erector spinae plane block
CRF: Case report form
LAs: Local anesthetics
